# Foreign Correspondence

**Published:** 1857-06

**Authors:** W. F. Westmoreland

**Affiliations:** Professor of the Principles and Practice of Surgery in the Atlanta Medical College


### ARTICLE II.

### FOREIGN CORRESPONDENCE.

By W. F. WESTMORELAND, M. D., Professor of the Principles and Practice of Surgery in the Atlanta Medical College.

PARIS, FEBRUARY 25TH, 1857.

*Dear Doctor*—The new anesthetic *amylène*, which for a month or two past has created some excitement in England, was, this morning, for the first time, administered in the hospitals of Paris. The numerous anesthetics that have been proposed, and found worthless,and in some instances extremely noxious, since the discovery of the anesthetic properties of chloroform and ether, has had the effect of rendering surgeons but little disposed to give credit, or to experiment with any new agents that may be proposed to produce insensibility, consequently the length of time that has elapsed since the discovery of the anesthetic properties of amylene and its introduction into the Profession, by Dr. Snow of London, and its first administration, by the surgeons of the hospitals of Paris, who as you are apprised, are notorious for their early experiments with all new remedies.

The chemical properties of amylene were discovered by M. Ballard in 1844. It is procured from an alcohol which is the result of the fermentation of the cuticle of the potato, and is known to French chemists under the name of *alcool amylique*. Dr. John Snow of London, as above suggested, has the honor of discovering its anesthetic properties, after various experiments upon animals and himself, he on the 10th November, 1856 administered it to two patients, but with only partial success. In December, he administered it in several cases for slight operations with complete success. On the 18th

December, Ferguson, of London, amputated a thigh, and operated for Stone with the patients, under the influence of amylene—the experiments in both cases were satisfactory. The 10th January, 1857, Dr. Snow made known his discovery to the Royal Society of London, reporting, at that time, twenty-two cases in which amylene had been administered. In this report Dr. Snow, gives the following as the principal facts that he had observed in the administration of this agent. The time necessary to produce insensibility varies from two to six minutes; the manner of administering it differs but little from that of chloroform. The quantity necessary to produce insensibility varies from half an ounce to two ounces. He has not observed in any case, that profuse salivation and nausea so constantly observed in the administration of chloroform; in some of the cases he has observed a rigidity of the muscles: in two cases, although there was complete insensibility, the intelligence of the patients presisted; Coma, when present, is not so profound as in the administration of chloroform. The circulation and respiration are accelerated.

The experiment this morning was at the clinic of M. Nelaton in presence of quite a number of physicians and students. The patient, a girl from 12 to 15 years ot age, had for some time suffered from necrosis of the tibia—general health apparently good. The same rules were observed in the administration of the amylene as are usual in the administration of chloroform. I suppose from an ounce and a half to two ounces were administered; the time required to produce insensibility was from five to eight minutes. There was no coma, no nausea or salivation with rigidity of the muscles. The patient, during the operation, which required several minutes, was perfectly quiet, eyes open, and a portion of the time conversed with those around the bed, without, apparently, being conscious of the operation which was going on. Being questioned immediately after the operation as to whether she experienced any pain, she replied without hesitation, that she had felt no pain. She complained of slight head-ache after the operation, but did not present that stupid appearance that is usually observed after the administration of chloroform. The experiment was, by all, pronounced satisfactory; but from one case, however satisfactory, little can be said, as the numerous experiments that we will likely have in the hospitals of Paris within the next few weeks may change entirely the opinion of those who witnessed this experiment, and amylene with all its pretensions may, like many other new agents that have been vaunted for a time fall in the estimation of the profession to rise no more.

There are at present in the wards of M. Nelaton, two cases of Peri-uterine hematocele, a lesion which until very recently was little understood, and even at present there exists more than one opinion as regards its precise character. For some years past M. Nelaton has paid considerable attention to this affection, and to him is due the honor of first calling the attention of the profession to it.

Peri-uterine hemotocele is an accumulation of blood forming a tumor, which is situated between the rectum vagina and uterus, and may be readily detected by an examination per vagina. The tumor varies greatly in size; in some cases it is not larger than a hen's egg, while in others it fills the pelvis, and may be felt in the illiac regions, compressing the vagina in such cases to such an extent that it is barely possible to reach the os uteri by this canal. If the tumor is large, by introducing the index and middle-finger into the vagina, which in some cases as above suggested, is rendered difficult from the great compression, and with the other hand compression is made over that portion of the tumor projecting in the illiac region, fluctuation will be more or less evident. If the tumor is small, we may cause the same sensation, by compressing the tumor between the two index fingers, one in the rectum and the other in the vagina. These tumors are in all cases situated immediately behind the uterus, apparently occupying the recto-uterine fold of peritoneum.

Much has been said of the general health of the subjects attacked, their menstrual irregularities, the rational signs which characterize the lesion, &c., &c.—each essayist giving and interpreting the various symptoms as best supported his views of the precise character of the affection. In giving the symptoms that are observed in this disease or accident, I perhaps could not do better than give the history of one of the cases above alluded to, which M. Nelaton says presents the symptoms usually observed in this affection. The patient is a stout healthy looking woman of 35 or 36 years of age; menstruates regularly and without pain—has for many years enjoyed the most perfect health. Eight days ago, the seventh day after the last mentioned period, she was attacked with a slight pain, or rather an uneasiness, in the hypogastric, and left illiac regions. This uneasiness increased, and at the expiration of a few hours was so intense that she applied for medical aid. A warm bath was prescribed. While in the bath, all the symptoms were greatly aggravated, the pain becoming so intense that it was impossible for her to occupy any one position for more than a moment at a time. The pain, after the bath, was no longer confined to the hypogastric and illiac regions, but descended towards the pelvic outlet. The physician was again called, and prescribed anodynes, which relieved, to a very great extent, the sufferings of the patient. For eight days, sometimes suffering greatly, at other times comparatively free from pain, she remained under treatment at her residence. Upon her entry into the hospital she presented the following symptoms: But little arterial excitement, no more than could be readily accounted for by the suffering of the patient; no heat of skin or any great thirst—nothing, in fact, to indicate a symtomatic fever of any intensity—an important negative symptom in point of diagnosis. The pain upon her entry into the hospital was not so acute as she had suffered; she described it as a dull heavy pain, giving the sensation as if something was attempting to escape by the vagina, the intensity of the suffering being in the region of the sacrum and vagina. Upon an examination per vagina a large tumor was detected, situated between the rectum uterus and vagina, apparently occupying the recto-uterine fold of peritoneum. The vagina was considerably depressed, its cul-de-sac posteriorly, being entirely effaced; the uterus was slightly displaced upwards—the os uteri directed towards the symphysis pubis. The tumor could be felt in the left illiac region. By palpation, as above suggested, fluctuation was evident. By an examination per rectum, the same fluctuating tumor was detected; the displacement of the bowel, however, was not so great as that of the vagina, neither were the parts so sensitive.

Such were the symptoms presented in this case, and are those usually observed in the affection. We have here an accumulation of blood—a bloody tumor, situated between the rectum, vagina and uterus. What is the origin of the blood thus accumulated? In what tissues is it located? Many conflicting opinions have been given in answer to the above questions, as to the precise character of the lesion, more, certainly, than I can possibly notice in this letter, as it was, alone, my purpose in commencing this article, to record the views of M. Nelaton, which, as will be seen, has under-went an entire change within the past two or three years.

The first investigations of M. Nelaton, led him to regard this affection as a kind of vicarious menstruation; that the accumulated blood was nothing more than the menstrual fluid. His theory, in a few words, was, that the periodic hemorrhage, instead of taking place in the usual way from the internal surface of the uterus, was discharged from the external surface of this organ into the subserous cellular tissue; that the accumulated blood occupies the cellular tissue, uniting the cul-de-sac of peritoneum to the adjacent organs.

Since adopting the above theory, he has had an opportunity of making several post-mortem examinations of patients who have died of this affection, which has entirely changed his views as regards the character of the lesion. He has demonstrated by these dissections, that the accumulated blood occupies the recto-uterine fold of peritoneum, instead of the subserous cellular tissue, as he had supposed, thus rejecting the idea of its origin from the external surface of the uterus. By a careful dissection of the tumor, he has in several cases been able to demonstrate a rupture of the peritoneum covering the ovarys, from which it was evident from the appearance of the adjacent parts that the hemorrhage had place.

His present opinion then, is that peri-uterine hematocele is always the result of a lesion of the peritoneum; that the rupture is upon that portion of the membrane covering the ovarys, and is, perhaps, in all cases, the result of the escape of a G. graafian visicle. That the lesion producing the hemorrhage is from this cause, is rendered more probable from the fact that the accident usually occurs about the menstrual period. Since making the post mortem examinations above alluded to, M. Nelaton has been making investigations to determine the probable exciting cause of such hemorrhages from a rupture of the peritoneum, covering a mature visicle of the ovary—a rupture which cannot by any means be considered abnormal, as it occurs every menstrual period. He says, that in quite the majority of cases that he has examined since adopting the above views as to the character of the lesion, he has found that the first symptoms of the hemorrhage, as uneasiness, pain, &c., have commenced immediately after coition.

In the case, the symptoms of which I gave above, I neglected to mention, that the first symptoms made their appearance immediately after a fatiguing sexual intercourse. Although he regards coition as the most frequent exciting cause of this accident, he is of the opinion that there is a predisposition or a predisposing cause of which, at present, we are ignorant.

There are several lesions with which this accident may be confounded, as extra-uterine pregnancy, peri-uterine abcesses, some forms of ovarian cysts, &c. Attention, however, to the history of the case, the symptoms, and a careful examination per vagina, will generally be sufficient to make the distinction.

The treatment formerly adopted by M. Nelaton, was invariably to give exit to the accumulated blood by means of a puncture through the walls of the vagina; if, however, as was sometimes the case, a simple puncture was not sfficient to evacuate the tumor, he enlarged the incision by means of a lithotome caché, turning out the clot, if necessary, with the finger. He has for sometime past renounced this plan of treatment, regarding it as extremely hazardous in a number of cases; says that the incision may be followed by the introduction of air into the cavity of the tumor, from which we may have all the unpleasant symptoms that usually result from the introduction of air into such cavities. His practice now is, to leave the tumor untouched; contending that in the majority of cases, if the patient be kept quiet, the blood will be absorbed. If, however, from some cause, the suffering of the patient is very great, or the tumor threatens to discharge its contents into the rectum, a puncture is indicated, which should always be practiced through the walls of the vagina.

The case above alluded to has now been in the hospital seven or eight days, and although the patient has at times suffered greatly, the treatment has consisted in the administration of such remedies as would tend to keep the patient quiet. The second case—the tumor is small, has never produced any great pain, and is at present, ten days after the first symptoms of the accident, considerably reduced in size.

---

PARIS, MARCH 13TH, 1857.

*Dear Doctor*—Since my letter of the 25th ultimo, in which I recorded the first experiment with amylene in the hospitals of Paris, quite the majority of surgeons of the public charities of the city have administered this agent; not, however, with the same success, as we find the most conflicting reports as to its effects. There are some who have in every case met with the most perfect success, while others have never been able to produce anesthesia—contending that it is an agent not to be relied upon—that while in some cases we may have the most complete success, in others it is entirely inert. Others, again, contend that from its odor, it is extremely disgusting to the patient, and that from its extreme volatility the surgeon and assistants are as likely to be effected as the patient.

M. Giraldes one of the surgeons to the *Hopital des Enfans Malades*, has perhaps experimented more extensively than any other surgeon in Paris. In a report to the *Societé de Chirurgie* a few days ago, he spoke in rather flattering terms of the agent,—says that he has administered amylene to twenty-five patients, and with the exception of one case, anesthesia was prompt. The patients were from three months to ten years of age; they all inhaled the agent without any great resistance; and in every case respiration was calm and normal. Anesthesia in every case was obtained without convulsions, nausea or vomiting, although some of the patients to which it was administered had but a short time before taken food. The duration of insensibility varied from one to three minutes. The patients were readily aroused, and, as in the case reported in my last letter, did not present that stupid or sluggish appearance so constantly observed, after the administration of chloroform.

M. Giraldes accounts for many of failures, and the large amount said to have been employed in several cases before producing anesthesia, to the manner of its administration. He says that amylene is much more volatile than chloroform, and if administered upon a compress, as the latter is usually administered in the hospitals of Paris, the air around the bed is impregnated with the vapor, and sometimes to such an extent, as above suggested, as to incommode seriously the assistants—the patient under such circumstances, inhaling but little if any of the agent. It should be administered so as to prevent the escape of the vapor. He has in all cases withdrawn the agent as soon as anesthesia was produced, consequently cannot judge of the length of time that it may be prolonged, without inconvenience. M. Giraldes is, however, of the opinion that it may be administered in all cases where it is desirable to produce anesthesia. As to its advantages, if any it has, over chloroform, he reserves his decision.

M. Nelaton presented a patient at his clinic a few days since which he six years treated for a white swelling of the hip joint, a disease usually described under that elastic term coralgia. His object, he said, in exhibiting this patient was to demonstrate the possibility of cure, in this terrible affection, and in speaking of a cure here, he said he had no reference to the usual terminations of the disease in cases where patients were fortunate enough to escape with their lives, in which we have either a dislocation of the femur of an anchylosis of the joint, but a cure in which all the functions of the limb are restored. The patient presented no deformity, whatever, of the hip-joint, it being evident that its functions were perfectly restored—the patient performing the same movements, and apparently with the same facility, with the limb in which the disease had existed as with that of the opposite. M. Nelaton contended that there was not the least doubt as to the character of the disease as by the symptoms, which were well marked, as well as other circumstances connected with the history of the case, it was readily distinguished from that less grave affection chronic arthritis, or other lesions with which this disease is sometimes confounded.

The plan of treatment adopted in this case was extensive cauterizations, over the diseased joint with the red-hot iron. M. Nelaton says that this is by no means the only case in which he has seen such a favorable result. That he has, for sometime been convinced that the disease is perfectly curable if the proper remedies be applied sufficiently early in the disease. He relies for success upon the actual cautery with such constitutional remedies as may be indicated. Says that the treatment, if successful, must be instituted early in the disease—that if we wait until there is a disorganization of the synovial membrane, softening of the cartilages, &c., before commencing the treatment, that we will invariably have either a dislocation of the head of the femur, or a partial or complete anclylosis of the joint. Says that he knows that there are surgeons, who object to the actual cautery in the commencement of the disease, regarding it as unnecessarily heroic. He is also apprised of the great difficulty in a number of cases of inducing the patient to submit to a plan of treatment, which they regard with such horror. Experience, whoever, has convinced him that it is the only remedy upon which we can rely, and even this, if deferred until the disease is considerably advanced, adopting it as a last resort as is counselled by some surgeons, will not be sufficient to arrest the progress of the disease.

The actual cautery is regarded by M. Nelaton as an important remedial agent, not only in the chronic affections of the joints, but in various other lesions.

He says that a great error is frequently committed in the application of this agent—an error which has had a tendency to bring the remedy in some disrepute. There are many who imagine that the sufferings, &c., of the patient, will be in proportion to the extent of the eschar—that a superficial cauterization will produce much less pain than one sufficiently extensive to produce an eschar, the thickness of the true skin. The reverse is true—superficial cauterization producing much more pain without any great benefit to the disease for which it is applied. He contends for the heroic application of the cautery, particularly in the affection above alluded to, producing an eschar, which sometimes extends to the sub-cutaneous cellular tissue. The location from such a cauterization which at first a little unsightly will, he contends disappear in a few years.

In a letter a few months ago, I spoke of a case of tertiary syphilis, in the words of M. Nelatan, which was being submitted, to the new plan of treatment known as *syphilization*, and promised to keep you informed, of the result of this novel treatment. There was at first, as I believe I informed you, an evident improvement in some of the symptoms—the sufferings of the patient which were at night very great, entirely disappearing—the ulcerations at the same time presenting a more healthy appearance.

There has been but little if any change in any of the symptoms since my last report, which was, if I am not mistaken, about the termination of the first series of inoculations, or when the pus obtained from the first inoculation would no longer produce a pustule. The second series of inoculations was continued only three weeks or a month, the patient from some cause refusing to submit to the treatment. I learn, however, from M. Auzaîs Teurenne who is conducting the experiment, that the inoculations will be re-commenced in a few days. The treatment has so far been conducted without any great inconvenience to the patient, although there has been as many as an hundred chancres upon the chest and superior extremities at one time. The marks the result of the chancres are only perceptible upon a careful examination.

Yours, &c.,
W. F. WESTMORELAND.

